# A biomimetic urchin-like photothermal nanoplatform for the efficient eradication of *Streptococcus mutans* and biofilm dispersion

**DOI:** 10.3389/fcimb.2026.1917604

**Published:** 2026-09-03

**Authors:** Yuxia Wang, Jing Mi, Xiaoling Cao, Muyao Wang, Huanhuan Feng, Fanying Kong, Xinge Zhang, Xiaobin Liu

**Affiliations:** 1 Department of Cariology and Endodontics, Tianjin Stomatological Hospital, School of Medicine, Nankai University, Tianjin, China; 2 Tianjin Key Laboratory of Oral and Maxillofacial Function Reconstruction, Tianjin Clinical Research Center for Oral Diseases, Tianjin Stomatological Hospital, Tianjin, China; 3 Department of Oral and Maxillofacial Surgery, Tianjin Stomatological Hospital, School of Medicine, Nankai University, Tianjin, China; 4 Key Laboratory of Functional Polymer Materials of Ministry of Education, Institute of Polymer Chemistry, Tianjin Key Laboratory of Functional Polymer Materials, College of Chemistry, Nankai University, Tianjin, China

**Keywords:** biomimetic nanomaterial, oral biofilm elimination, photothermal therapy, sea urchin-like gold nanoparticles, *Streptococcus mutans*

## Abstract

**Background:**

Dental caries, primarily driven by *Streptococcus mutans* (*S. mutans*) biofilms, remains a formidable clinical challenge due to the protective extracellular polymeric substance (EPS) matrix and the limited efficacy of conventional antibiotics.

**Methods:**

To address this, we report a biomimetic photothermal nanoplatform (FWA NPs) featuring a hierarchical, urchin-like multi-spiked architecture, synthesized via the co-assembly of ferrocene-tryptophan conjugates and in situ biomineralized gold nanoparticles.

**Results:**

Benefiting from this unique topological structure, FWA NPs maximize interfacial interactions with bacterial membranes and exhibit enhanced near-infrared absorption, achieving a remarkable photothermal conversion efficiency of 54.4%. Under 808 nm near-infrared (NIR) irradiation, this rapid and localized heat generation induced efficient eradication of *S. mutans*, which manifested as a dramatic reduction in bacterial colonies from ~107 to ~105 CFU/mL and irreversible membrane damage characterized by massive surface wrinkling, localized collapse, and membrane rupture. Additionally, FWA NPs reduced the survival rate of *S. mutans* biofilms to approximately 10%, Crucially, FWA NPs demonstrated excellent biocompatibility with a hemolysis rate of approximately 1% for red blood cells and a relative survival rate of 90% for normal cells.

**Conclusion:**

By synergistically integrating topological advantages with highly efficient energy conversion, this rationally designed nanoplatform offers a highly effective, non-antibiotic therapeutic paradigm for combating biofilm-associated oral infections and mitigating the global threat of antimicrobial resistance.

## Introduction

As a ubiquitous global chronic disease, dental caries severely compromises oral health and imposes a substantial socioeconomic burden on public health systems (do et al., 2025; Chen et al., 2025a; Li et al., 2025). While the discovery of antibiotics has equipped humanity with a potent arsenal against various infections, the excessive and inappropriate administration of these drugs has accelerated the evolution of bacterial resistance, thereby spurring the emergence of drug-resistant strains (Zampieri., 2021; Theuretzbacher, 2024). Exacerbated by poor oral hygiene and high-carbohydrate diets, biofilm formation further compromises the therapeutic efficacy of conventional antibiotics (Chen et al., 2024; Tuganbaev et al., 2022). For example, *Streptococcus mutans* (*S. mutans*), a primary cariogenic pathogen, can adhere to the pellicle-coated enamel surface via highly specific adhesin-carbohydrate interactions (Xu et al., 2026). Subsequently, these bacteria metabolize carbohydrates into extracellular polymeric substances (EPS), embedding themselves within this self-produced matrix to sustain a viable microbial consortium (Tong et al., 2026; Sharma et al., 2023). The resulting EPS matrix-composed of diverse macromolecules such as exopolysaccharides, proteins, and extracellular DNA-functions as a formidable protective scaffold. It not only shields *S. mutans* from the host’s innate immune clearance but also severely restricts the intra-biofilm penetration of antibiotics (Bowen et al., 2018; Rumbaugh and Sauer, 2020). Therefore, there is an urgent need for the development of innovative, anti-infective therapeutics that do not induce drug resistance.

Near-infrared (NIR) light-mediated phototherapy has garnered considerable attention as a viable strategy to combat bacterial infections, largely owing to its distinct advantages, including spatiotemporal controllability, minimal invasiveness, adequate tissue penetration depth, and the ability to circumvent bacterial resistance mechanisms (Chen et al., 2025b; Zhu et al., 2026). In photothermal therapy (PTT), localized hyperthermia generated by photothermal conversion agents under NIR irradiation effectively eradicates bacteria through multiple mechanisms: inducing protein denaturation, causing irreversible cellular damage, and disrupting cell membrane integrity which leads to concomitant increase in permeability (Wang et al., 2026; Kim et al., 2026). However, conventional photothermal agents, particularly small-molecule organic dyes, still suffer from several inherent limitations in antibacterial applications. For instance, indocyanine green, a representative NIR dye, often exhibits poor photostability, susceptibility to photobleaching, a tendency to aggregate in aqueous media, rapid degradation, and a short *in vivo* half-life. These drawbacks cause insufficient sustained heat generation and limited retention of the agent at the infection site, severely compromising its antibacterial efficacy in complex infection microenvironments.

Furthermore, dense biofilms and their associated EPS matrices at oral infection sites severely hinder the deep diffusion and intra-biofilm penetration of conventional photothermal agents (Sharma et al., 2023). This limited penetration results in heterogeneous local heating, which often requires higher laser power densities or prolonged irradiation times to achieve ideal bactericidal effects. Paradoxically, this increases the risk of non-specific thermal damage to surrounding healthy tissues.

In contrast, urchin-like nanoparticles, characterized by their multi-spiked, rough, and hierarchical surface topographies, can induce a pronounced localized electromagnetic field enhancement under NIR irradiation. Additionally, their unique morphology significantly enhances light-harvesting capability and photothermal conversion efficiency through multiple light scattering events and a substantially expanded surface area (Qin et al., 2024; Eker et al., 2024). Their distinctive architectures also afford abundant surface active sites and an enlarged interior capacity for drug loading. This structural advantage facilitates the encapsulation of conventional small-molecule photothermal and antibacterial compounds, thereby mitigating their inherent tendency to aggregation, premature inactivation, and inadequate local retention. More importantly, the multi-spiked topography of urchin-like structures forms a larger contact interface with bacteria and biofilms. Some urchin-like nanosystems can even exert physical disruption on biofilms. This not only promotes the deep penetration of thermal effects into the biofilm matrix but also dramatically improves the overall antibacterial efficacy (Delgado-Corrales et al., 2025). Accordingly, the developing of urchin-like photothermal nanoplatforms has great potential for achieving improved bactericidal and biofilm-eradicating performance under milder treatment conditions or lower laser doses, representing a promising strategy for the management of oral infectious diseases.

**Figure 1:**
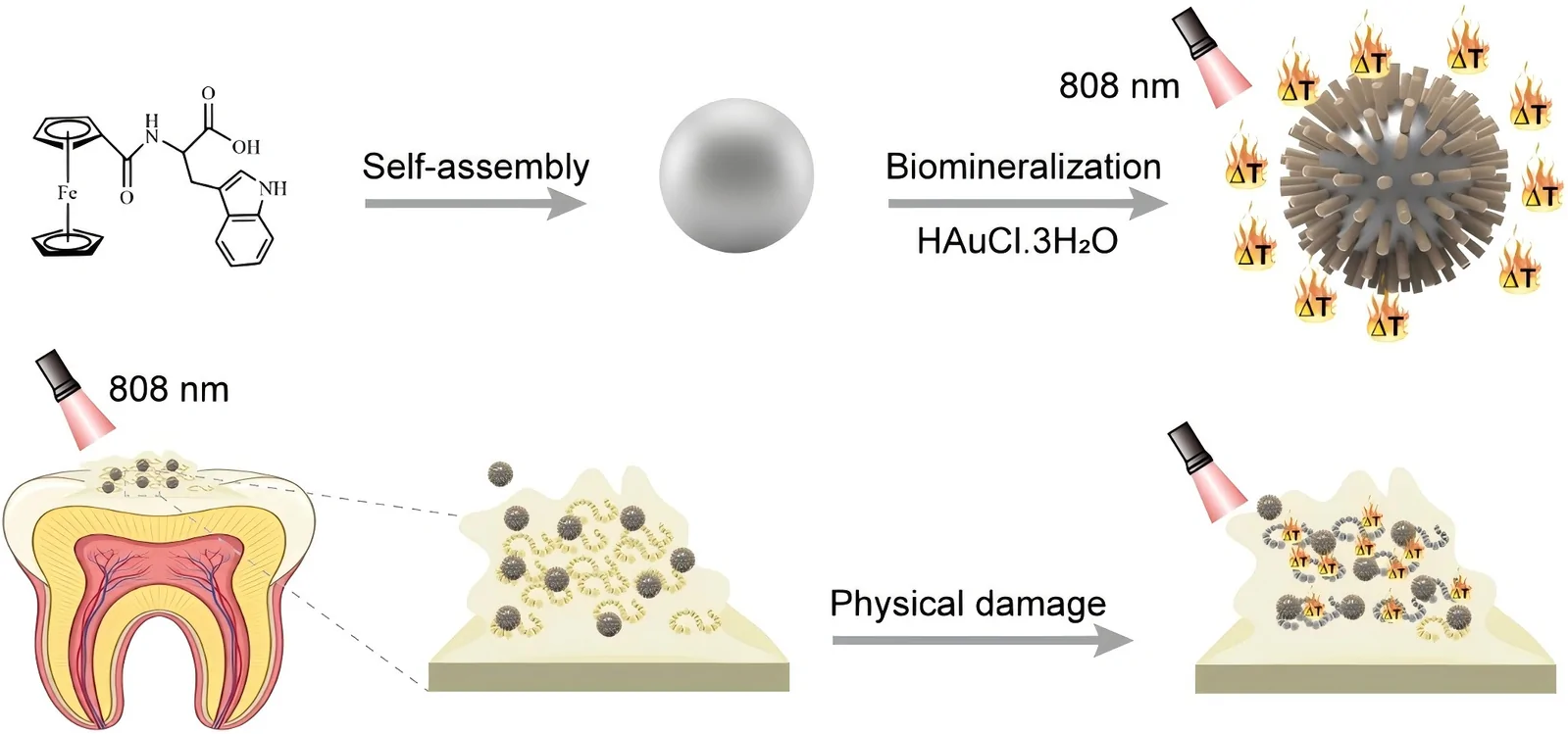
Schematic representation of the construction of sea urchin-like FWA NPs and their synergistic photothermal mechanisms for bacterial eradication and biofilm dispersion under NIR irradiation.

To address these challenges, inspired by the architecture of sea urchins, we engineered a biomimetic photothermal nanoplatform comprising ferrocene-tryptophan (Fc-W) conjugates and gold nanoparticles (Au NPs). As illustrated in **Figure 1**, the Fc-W conjugates initially undergo a hierarchical self-assembly process to form stable nanoaggregates. Subsequently, driven synergistically by the redox properties of the ferrocene moieties and the reducing capability of tryptophan residues, Au NPs are generated *in situ* and grow directionally on the aggregate surface. This process ultimately yields urchin-like self-assembled nanoparticles (FWA NPs) with a multi-spiked, hierarchical surface topography. Benefiting from their unique morphological features, FWA NPs exhibit significantly enhanced near-infrared (NIR) light absorption and excellent photothermal conversion efficiency. At the same time, this distinctive architecture maximizes the interface contact area with both bacteria and biofilms, thereby improving the local photothermal bactericidal efficacy. Upon NIR irradiation, the localized hyperthermia generated by the FWA NPs severely disrupts the structural integrity of the bacterial cell membranes and increase membrane permeability, leading to the efficient eradication of both planktonic and biofilm-associated bacteria. This fabrication strategy for the urchin-like nanoplatform not only provides an innovative paradigm for the synergistic co-assembly of functional organic small molecules and inorganic nanomaterials but also offers a promising therapeutic approach for the clinical treatment of oral infections and recalcitrant biofilm-associated diseases.

## Materials and methods

### Materials

L-Tryptophan methyl ester hydrochloride (98%), ferrocenecarboxylic acid (Fc-COOH, 98%), gold(III) chloride trihydrate (HAuCl_4_·3H_2_O, Au 49.5% on metal basis), β-D-galactose pentaacetate (98%), methacryloyl chloride, and 1-hydroxybenzotriazole (HOBt) were purchased from Aladdin (Shanghai, China). 1-Ethyl-3-(3-dimethylaminopropyl) carbodiimide hydrochloride (EDC), methanol (MeOH), dichloromethane (DCM), 2-methylpropionyl chloride, and triethylamine (TEA) were obtained from Sinopharm Chemical Reagent Co., Ltd. (Shanghai, China). Prior to use, liquid organic reagents and solvents (including MeOH, DCM, 2-methylpropionyl chloride, and TEA) were dried over calcium hydride and distilled. Dulbecco’s Modified Eagle Medium (DMEM), fetal bovine serum (FBS), trypsin, non-essential amino acids, and penicillin/streptomycin solution were purchased from J&K Scientific Ltd. (Beijing, China). All other chemicals were obtained from commercial suppliers. Where applicable, all organic solvents used in the syntheses were strictly dehydrated and dried before use.

### Synthesis of Fc-W-OMe

All reactions were performed under an anhydrous nitrogen atmosphere unless otherwise noted. Ferrocenecarboxylic acid (690 mg, 3.0 mM) was dissolved in anhydrous dichloromethane (DCM, 20 mL). Under an ice-water bath, triethylamine (TEA, 1.5 mL, 10.8 mM) was added dropwise to the solution, and the mixture was stirred for 1 h to activate the carboxyl group. Subsequently, 1-hydroxybenzotriazole (HOBt, 810.8 mg, 6.0 mM) and 1-ethyl-3-(3-dimethylaminopropyl)carbodiimide (EDC, 1152 mg, 6.0 mM) were added, and the reaction was allowed to proceed for 1 h. Finally, L-tryptophan methyl ester hydrochloride (H-Trp-OMe·HCl, 762 mg, 3.0 mM) was introduced into the mixture. The resulting reaction mixture was stirred in a water bath at 25°C for 2 h. Upon completion, the organic mixture was washed successively with aqueous NaHCO_3_ (1 M, 3 × 50 mL), aqueous HCl (1 M, 3 × 50 mL), and deionized water (3 × 100 mL). The organic layer was dried over anhydrous Na_2_SO_4_ and filtered. The solvent was completely removed under reduced pressure at 39°C to yield the intermediate Fc-W-OMe as an orange crystalline solid (817.5 mg, yield: 56.3%), which was used in the next step without further purification.

### Synthesis of Fc-W (deprotection of Fc-W-OMe)

The deprotection of Fc-W-OMe was performed according to a previously reported procedure (Yang et al., 2019)^]^. Briefly, Fc-W-OMe (466 mg, 1.0 mM) was dissolved in methanol (MeOH, 10 mL). An aqueous NaOH solution (1 M, 10 mL) was slowly added to the mixture, which was then stirred for 12 h. After the reaction was completed, aqueous HCl (0.1 M) was added to quench the reaction and neutralize the solution, followed by the removal of MeOH under reduced pressure at 39°C. The remaining residue was redissolved in DCM and washed successively with deionized water (3 × 50 mL), aqueous NaHCO_3_ (1 M, 3 × 50 mL), aqueous HCl (1 M, 3 × 50 mL), saturated brine, and deionized water (3 × 100 mL). The combined organic extracts were dried over anhydrous Na_2_SO_4_, filtered, and concentrated under reduced pressure at 39°C. The crude product was finally purified by flash column chromatography using a gradient eluent of DCM/MeOH (20:1, v/v) to afford the target compound Fc-W. Yield: 62.1% (258.5 mg).

### Preparation of FWA NPs

Briefly, 4 mg of Fc-W powder was completely dissolved in 500 µL of DMSO to obtain a clear solution. Under vigorous magnetic stirring, this solution was injected dropwise via a microsyringe into a 3.5 mL Schering vial containing deionized water. The mixture was continuously stirred for 15 min to drive the self-assembly of Fc-W molecules, leading to the formation of the initial nanoparticle suspension. Subsequently, the suspension was transferred into a round-bottom flask and maintained under steady stirring at room temperature. A pre-mixed solution comprising 500 µL of 1% Tween 20 solution and 1 mL of aqueous HAuCl_4_·3H_2_O (2 mg/mL) was then added dropwise into the above suspension. The reaction mixture was incubated at 37 °C under constant shaking for 3 h, facilitating the *in situ* reduction and deposition of gold nanoparticles to produce FWA NPs. Finally, the resulting FWA NPs were collected by centrifugation at 12,000 rpm for 10 min. The obtained pellet was washed and thoroughly redispersed in deionized water via mild sonication to afford the purified FWA NPs suspension.

### Hemolysis assay

To evaluate the hemocompatibility of FWA NPs, an *in vitro* hemolysis assay was conducted using fresh human blood samples. Briefly, the collected whole blood was centrifuged at 3000 rpm for 10 min to isolate red blood cells (RBCs). The obtained RBCs were carefully washed five times with PBS to thoroughly remove plasma and other impurities. The purified RBCs were then resuspended in PBS to prepare a 5% (v/v) RBC suspension. Subsequently, the RBC suspension was mixed with equal volumes of FWA NPs solutions at various concentrations and incubated at 37°C for 1 h. Mixtures treated with an equal volume of PBS and 1% Triton X-100 served as the negative and positive controls, respectively. After incubation, the suspensions were centrifuged, and the resulting supernatants were carefully transferred into a 96-well plate. The absorbance of the released hemoglobin in the supernatants was recorded at 545 nm using a microplate reader. The hemolysis rate of each sample was calculated according to the following equation:


$$\%\text{ Hemolysis}=\;\frac{{\text{A}}_{sample}-{\text{A}}_{negative\text{ control}}}{{\text{A}}_{positive\;control}-{\text{A}}_{negative\;\text{control }}}\times 100\%$$

Where A*_sample_*, A*_negative control_*, and A*_positive control_* represent the absorbance of the experimental group, negative control, and positive control, respectively.

### Cytotoxicity assay

The *in vitro* cytotoxicity of FWA NPs was evaluated on human oral keratinocytes (HOK cells) using a standard Cell Counting Kit-8 (CCK-8) assay. Briefly, HOK cells were seeded into 96-well plates at a density of 1 × 10^4^ cells per well and cultured in a humidified incubator (37 °C, 5% CO_2_) for 24 h to allow for complete cell attachment. Subsequently, the original culture medium was removed and replaced with fresh medium containing various concentrations of FWA NPs. The cells were then incubated for an additional 24 h. Following the co-incubation period, 10 μL of CCK-8 working solution was added to each well, and the plates were further incubated in the dark for 1 h. The absorbance of each well at a wavelength of 450 nm was measured using a microplate reader. Cells cultured in the fresh medium without FWA NPs served as the control group, while wells containing only the medium and CCK-8 reagent without cells served as the blank. The relative cell viability of each sample was calculated according to the following equation:


$$\%\text{Cell viability}=\frac{{\text{A}}_{sample}-{\text{A}}_{blank\;}}{{\text{A}}_{positive\;control}-{\text{A}}_{blank\;}}\times 100\%$$

Where A*_sample_*, A*_positive control_*, and A*_blank_* represent the absorbance of the FWA NPs-treated group, the untreated control group, and the blank wells, respectively.

### Antibacterial test

The *in vitro* photothermal antibacterial activity of FWA NPs was evaluated using *S. mutans* as the model bacterium. Briefly, *S. mutans* in the logarithmic growth phase was diluted to a concentration of approximately 10^6^ CFU/mL. The bacterial suspension was then mixed with varying concentrations of FWA NPs and pre-incubated at 37°C for a predetermined period. Subsequently, the experimental mixtures were exposed to an 808 nm NIR laser irradiation for 10 min, while the parallel groups without NIR irradiation served as the dark controls. Following the treatments, the bacterial suspensions were serially diluted with PBS, and appropriate aliquots were spread onto Brain Heart Infusion agar plates. After incubation at 37°C for 24 h, the colony-forming units were quantified to evaluate the bactericidal efficacy of the FWA NPs.

To further elucidate the morphological and structural alterations of the bacteria post-treatment, the treated bacterial suspensions were centrifuged to harvest the cell pellets. The collected bacterial cells were washed with PBS and fixed with 2.5% glutaraldehyde. The fixed samples were then subjected to sequential dehydration through a graded ethanol series. Afterwards, the dehydrated bacterial suspensions were drop-cast onto silicon wafers, dried, and sputter-coated with gold. The surface morphology and structural integrity of the bacteria were ultimately observed using SEM.

### Biofilm dispersion assay

To evaluate biofilm dispersion, *S. mutans* suspension (OD_600_ = 0.025 in BHI broth with 0.5% maltose) was seeded into 96-well plates (120 μL/well) and statically incubated at 37°C for 24 h. After washing with PBS to remove planktonic cells, the mature biofilms were treated with various concentrations of FWA NPs (PBS as control) for 4 h. For photothermal groups, samples were subsequently irradiated with an 808 nm NIR laser for 5 min. Post-treatment, the residual biofilms were washed, fixed with absolute methanol (15 min), and stained with 0.5% (w/v) crystal violet (15 min). After rinsing off unbound dye and air-drying, the biofilm-bound dye was solubilized with 33% (v/v) glacial acetic acid under gentle shaking for 10 min. Finally, the absorbance at 590 nm was measured via a microplate reader to quantify the residual biofilm biomass.

### Statistical analysis

All experiments were performed in triplicate and results are representative of at least three independent experiments. Comparisons between groups were analyzed by t-test. Data are expressed as means ± SE and results were considered to be statistically significant where *p < 0.05*.

## Results

### Synthesis and characterization of FWA NPs

**Figure 2:**
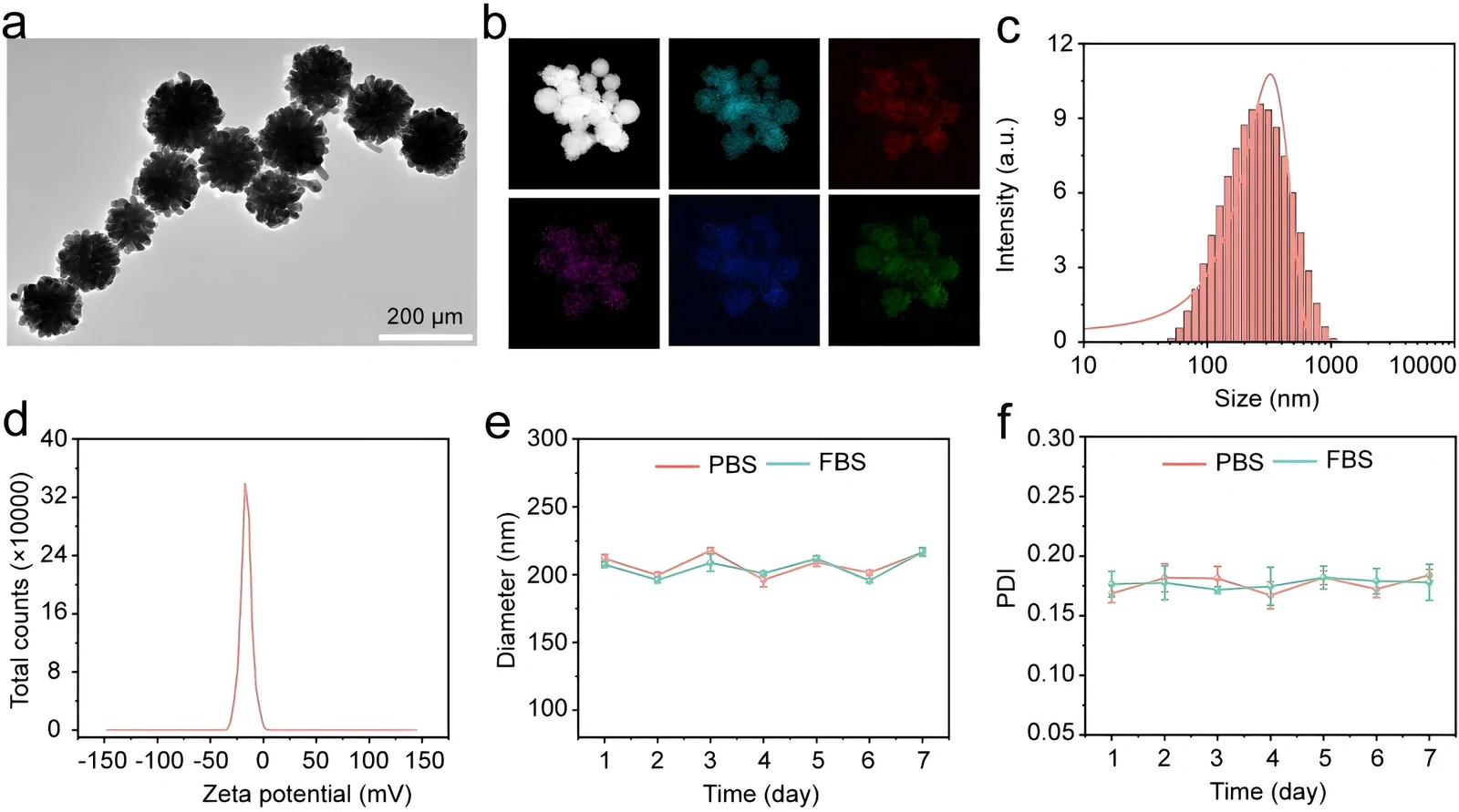
Morphology, elemental composition, and stability characterization of FWA NPs. **(a)** Transmission electron microscopy (TEM) image of FWA NPs. **(b)** Corresponding elemental mapping images, demonstrating the uniform distribution of constituent elements within the nanoparticles. **(c)** Hydrodynamic size distribution measured by DLS. **(d)** Zeta potential distribution. **(e, f)** Changes in the hydrodynamic size and polydispersity index of FWA NPs over a 7-day incubation period in PBS and FBS, respectively.

To evaluate the structural characteristics and physicochemical properties of FWA NPs, systematic characterization was performed. As shown in **Figure 2A**, transmission electron microscopy (TEM) images revealed that FWA NPs possess a highly uniform sea urchin-like nanostructure, characterized by distinct radial protrusions and a rough, hierarchical interface. Corresponding elemental mapping (**Figure 2B**) further confirmed that the constituent elements are uniformly distributed and highly overlapped within the nanoparticles, indicating the successful formation of a stable composite structure rather than simple physical mixing or localized aggregation. Furthermore, dynamic light scattering (DLS) analysis showed a relatively narrow and uniform hydrodynamic size distribution (**Figure 2C**), which, together with the sharp and symmetrical zeta potential peak (**Figure 2D**), demonstrates excellent electrostatic balance and interfacial consistency in the dispersed state. These properties effectively prevent disordered aggregation and ensure the synergistic action of various functional units at the bacteria-material interface.

Given that maintaining colloidal stability in complex physiological environments is a prerequisite for efficient antibacterial performance, we further investigated the long-term dispersion behavior of FWA NPs in phosphate-buffered saline (PBS) and fetal bovine serum (FBS). As illustrated in **Figures 2E, F**, the average hydrodynamic size of FWA NPs remained stable at approximately 200 nm throughout a 7-day incubation period, with the polydispersity index maintained at a consistently low level.

### Photodynamic performance of FWA NPs

**Figure 3:**
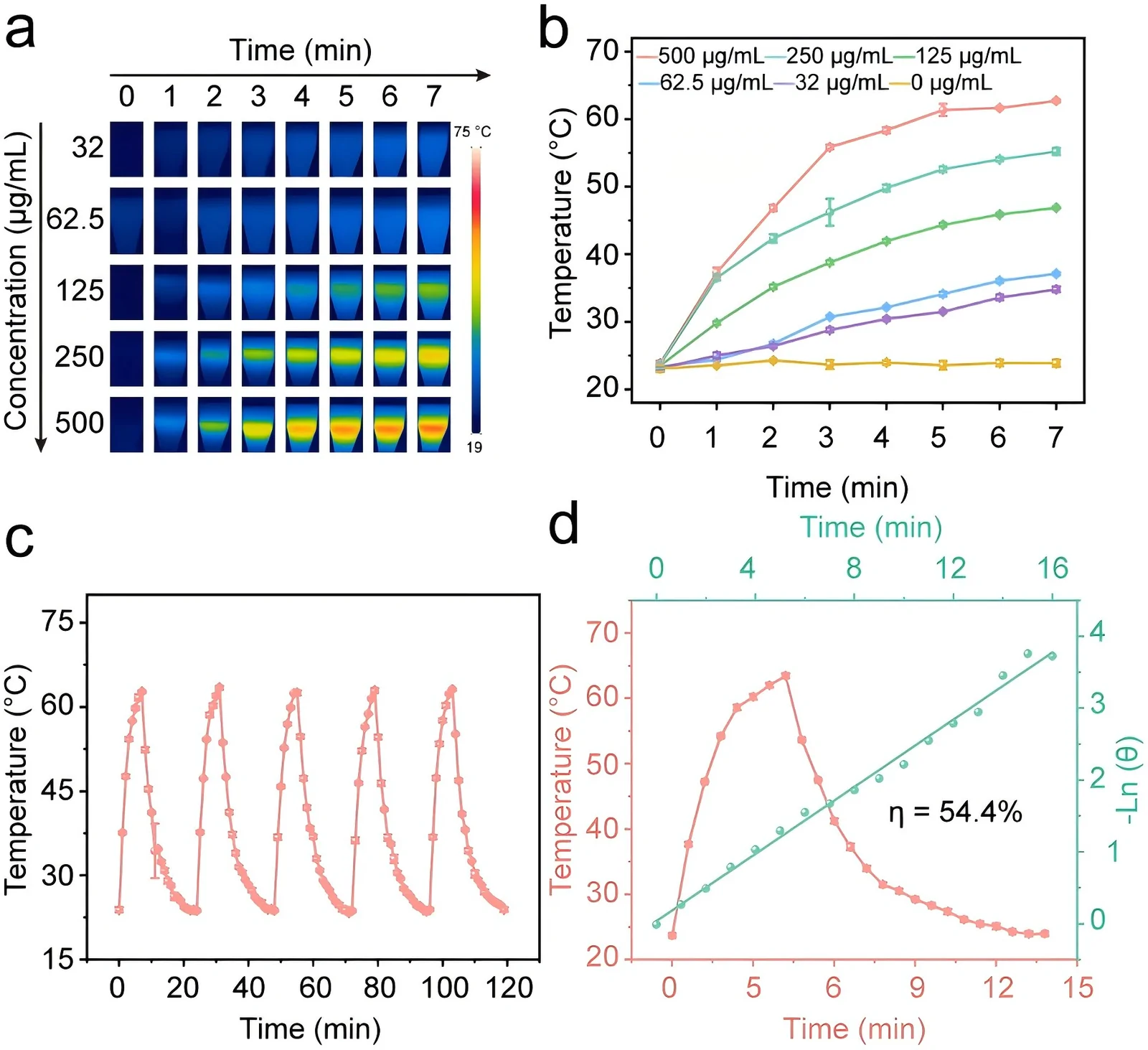
Photothermal characterization of FWA NPs. **(a)** Representative infrared thermal images of FWA NPs at various concentrations under 808 nm laser irradiation over a period of 7 min. **(b)** Temperature evolution curves of FWA NPs at different concentrations as a function of irradiation time. **(c)** Photothermal stability of FWA NPs over multiple laser on/off cycles. **(d)** Photothermal heating and natural cooling curves of FWA NPs, along with the linear fitting of the cooling period used for the calculation of photothermal conversion efficiency.

To systematically evaluate the photothermal performance of FWA NPs under 808 nm laser irradiation, we first monitored their concentration-dependent heating behavior. As shown in **Figures 3A, B**, both the heating rate and the maximum temperature of the solutions increased significantly as the nanoparticle concentration escalated from 32 to 500 μg/mL. At the highest tested concentration (500 μg/mL), the solution temperature rapidly surged from room temperature to over 60 °C within merely 7 min, demonstrating superior light-harvesting and heat-generating capabilities. Furthermore, five consecutive “laser on/off” cycling tests (**Figure 3C**) revealed that the peak temperatures and heating profiles remained almost perfectly consistent without obvious attenuation or response hysteresis, thereby verifying the excellent photothermal stability and robust photobleaching resistance of the FWA NPs. Subsequently, by recording the full heating and natural cooling processes and applying linear fitting to the cooling period (**Figure 3D**), the photothermal conversion efficiency (η) of this nanoplatform was calculated to be remarkably high at 54.4%.

### Photothermal eradication of *S. mutans* and its biofilms by FWA NPs

**Figure 4:**
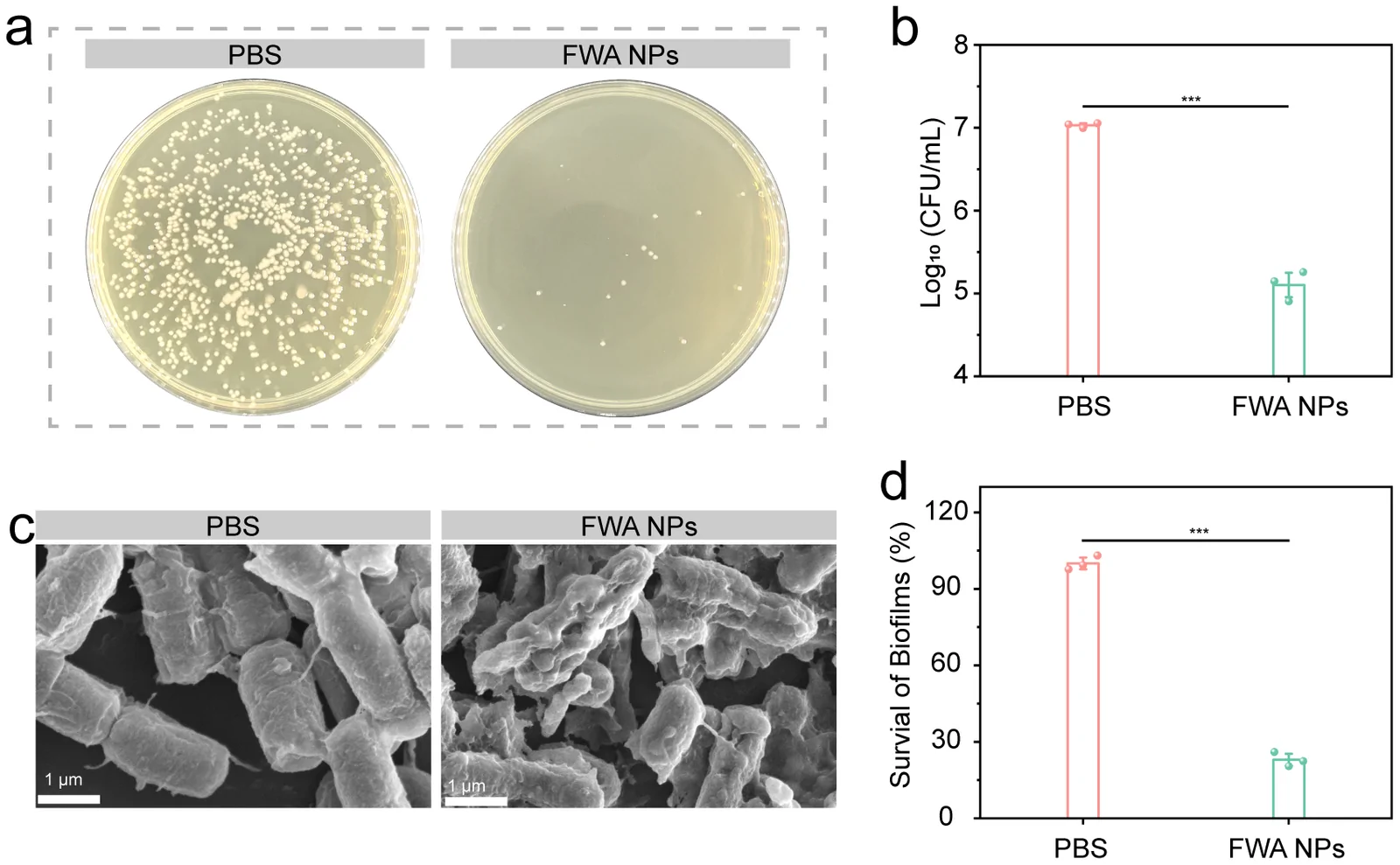
Photothermal antibacterial and biofilm dispersion performance of FWA NPs. **(a)** Representative photographs of bacterial colonies on agar plates for the PBS and FWA NPs-treated groups. **(b)** Corresponding statistical analysis of viable bacterial counts. **(c)** Representative SEM images revealing the morphological changes of bacteria following treatment with PBS and FWA NPs groups. **(d)** Quantitative evaluation of biofilm survival rates across the different treatment groups. The data are presented as the means ± standard errors, and the asterisks represent significant differences compared with the PBS group (*p < 0.05, **p < 0.01, ***p < 0.001).

A standard plate counting assay was performed and the result showed that the FWA NPs-treated group exhibited a dramatic reduction in bacterial colonies compared to the PBS group, which maintained dense colony formation (**Figure 4A**). Quantitative analysis further confirmed a significant decline in the bacterial burden, with the viable count dropping by approximately two orders of magnitude (from ~10^7^ to ~10^5^ CFU/mL) (**Figure 4B**). To elucidate the underlying bactericidal mechanism, the morphological alterations of *S. mutans* were examined using scanning electron microscopy (SEM). While the PBS-treated bacteria maintained their typical smooth and intact cellular outlines, bacteria exposed to FWA NPs displayed severe structural abnormalities, including massive surface wrinkling, localized collapse, and membrane rupture (**Figure 4C**). Recognizing that oral infections such as dental caries are primarily driven by resilient, mature biofilms rather than planktonic bacteria, we further investigated the antibiofilm performance of FWA NPs. As illustrated in **Figure 4D**, FWA NPs treatment plummeted the survival rate of *S. mutans* biofilms to approximately 10%, in stark contrast to the near 100% viability observed in the PBS group.

### *In vitro* biosafety evaluation of FWA NPs

**Figure 5:**
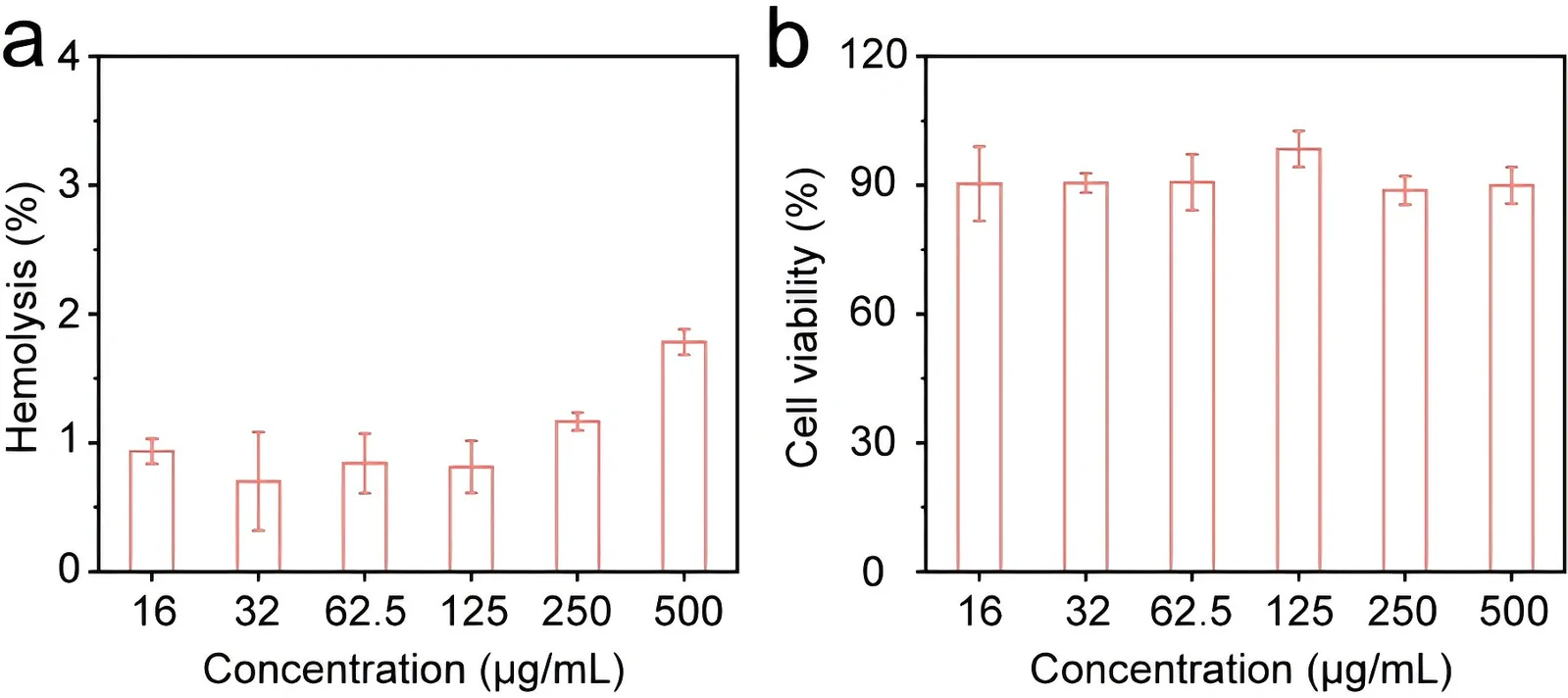
Hemocompatibility and cytocompatibility evaluation of FWA NPs. **(a)** Hemolysis rate of red blood cells treated with various concentrations of FWA NPs. **(b)** Relative cell viability after incubation with different concentrations of FWA NPs. The data are presented as the means ± standard errors, and no significant differences were found between groups.

Given that excellent biosafety is a prerequisite for the clinical translation of localized antibacterial nanomaterials, we systematically evaluated the *in vitro* hemocompatibility and cytocompatibility of FWA NPs. As shown in **Figure 5A**, the hemolysis rate of red blood cells incubated with FWA NPs at concentrations ranging from 16 to 500 μg mL^-1^ remained at an exceptionally low level (approximately 1%). Even at the highest tested concentration (500 μg mL^-1^), the hemolysis rate was well below the internationally recognized safe threshold of 5%. In the cell viability assays, following treatment with the same concentration gradient of FWA NPs, the relative survival rate of normal cells consistently remained around 90% without exhibiting any obvious dose-dependent cytotoxicity, confirming the absence of direct, non-specific toxicity to mammalian cells (**Figure 5B**).

## Discussion

The global prevalence of dental caries represents a significant public health concern. It not only causes a huge economic burden, but also affects individuals of all ages (GBD, 2026; Sun et al., 2026). *S. mutans* is one of the primary cariogenic pathogens in caries development (Xu et al., 2026). Conventional broad-spectrum antimicrobial approaches have limited application for caries prevention due to four key limitations: adverse side effects, impotent penetration into dense biofilms, the development of drug-resistant bacteria, and failure to disperse biofilms (Antimicrobial Resistance Collaborators, 2022). Nanoparticles have been recognized as a promising strategy for caries control. In this study, we engineered a biomimetic photothermal nanoplatform comprising ferrocene-tryptophan (Fc-W) conjugates and gold nanoparticles (Au NPs).

To develop a molecular building block capable of inducing the formation of sea urchin-like nanostructures, we first synthesized the intermediate Fc-W-OMe via a standard EDCI/HOBt coupling strategy using ferrocenecarboxylic acid and tryptophan methyl ester. Subsequently, the target compound, Fc-W, was successfully generated via alkaline hydrolysis deesterification (**Supplementary Figures 1**, **S2**). Notably, this small biomolecule covalently integrates a redox-active ferrocene moiety with a reducing tryptophan unit within a single molecule. This rational structural design lays the molecular foundation for its subsequent self-assembly behavior, as well as the *in situ* generation and directional growth of gold nanoparticles.

The unique morphology not only significantly increases the specific surface area and exposes abundant interfacial contact sites but is also expected to enhance physical topological interactions with bacterial cell membranes and biofilm matrices. Such a structure provides a robust foundation for localized heat focusing and the enrichment of active species during subsequent photothermal therapy. No significant fluctuations in size, aggregation, or precipitation were observed, indicating that FWA NPs can effectively resist structural instability induced by high ionic strength and complex protein adsorption in biologically relevant environments. This superior physiological stability ensures that the material maintains dispersion within actual infection microenvironments, thereby avoiding fluctuations in antibacterial efficacy and reductions in penetration capability caused by localized aggregation. Collectively, the unique sea urchin-like surface topology, uniform elemental distribution, and excellent stability in complex media highlight the significant potential of FWA NPs as a robust nanoplatform for antibacterial and antibiofilm applications.

The outstanding energy conversion efficiency (η = 54.4%) is closely attributed not only to the intrinsic optical properties of the material but also to its unique sea urchin-like rough interface, which likely enhances localized surface plasmon resonance and internal light scattering. From the perspective of localized anti-infection therapy, such a highly efficient, stable, and concentration-controllable photothermal effect holds immense translational value. On one hand, FWA NPs can rapidly exceed the critical temperature threshold required to induce bacterial protein denaturation and membrane disruption, facilitating the rapid eradication of *S. mutans*. On the other hand, the continuous and stable thermal stress output effectively degrades the dense EPS matrix of biofilms, triggering the irreversible disintegration of persistent bacterial communities. More importantly, such an exceptional photothermal conversion efficiency means that the desired bactericidal temperature can be achieved with a lower laser power density or a shorter irradiation time. This not only meets the clinical requirement for therapeutic efficacy but also reduces the risk of collateral thermal damage to fragile, healthy oral tissues, laying a solid biophysical foundation for the safe application of this photothermal platform (Wang et al., 2025).

The FWA NPs-treated group of *S. mutans* exhibited a dramatic reduction in bacterial colonies and viable counts, indicating the photothermal antibacterial efficacy of FWA NPs. An intact bacterial cytoplasmic membrane constitutes the basic biological structure required for bacterial viability (Strahl and Errington, 2017). In this study, the profound morphological changes indicated that the localized hyperthermia generated by FWA NPs under NIR irradiation irreversibly disrupts the integrity of the bacterial cell membrane, leading to the leakage of intracellular contents and subsequent cell death. When acting on *S. mutans* biofilms, FWA NPs also showed robust antibiofilm activity. This can be attributed to the unique sea urchin-like topography of FWA NPs; the multi-spiked rough interface provides an expansive contact area that facilitates strong adhesion to both the bacterial surface and the EPS matrix. Upon NIR activation, the focused thermal stress not only directly eradicates the embedded bacteria but also compromises the structural scaffold of the EPS, promoting the dispersion and disintegration of the mature biofilm. Collectively, this dual-action modality-simultaneously inflicting severe bacterial membrane damage and dismantling the protective biofilm architecture-demonstrates the substantial potential of FWA NPs as a highly effective photothermal nanoplatform for the management of intractable biofilm-associated oral diseases.

Hemocompatibility and cytocompatibility assays indicated that FWA NPs do not compromise erythrocyte membrane integrity, thereby minimizing the risk of hematological toxicity when they contact bleeding wounds or inflamed oral tissues. Subsequent cell viability assays further corroborated this favorable safety profile. Collectively, FWA NPs demonstrate remarkable physiological mildness and negligible toxicity. This characteristic is crucial for establishing the therapeutic rationale of our study: it provides compelling evidence that the robust bactericidal and biofilm-degrading capabilities of FWA NPs do not stem from inherent chemical toxicity, but are driven entirely by the precise, NIR-triggered localized photothermal effect. Given the anatomical complexity and tissue fragility of the oral microenvironment, an ideal therapeutic strategy must efficiently eradicate *S. mutans* while strictly avoiding collateral damage to surrounding healthy tissues. Therefore, the exceptional biosafety profile exhibited by FWA NPs perfectly aligns with the core demands of high selectivity and safety for localized anti-infection therapy, thereby laying a solid biological foundation for their clinical translation in the management of oral infections.

In summary, we have successfully engineered a biomimetic, urchin-like photothermal nanoplatform (FWA NPs) via the hierarchical co-assembly of ferrocene-tryptophan conjugates and the *in situ* biomineralization of gold nanoparticles. Benefiting from its unique multi-spiked topological architecture, FWA NPs exhibit significantly enhanced NIR absorption and an outstanding photothermal conversion efficiency of 54.4%. Under 808 nm irradiation, the rapid and localized heat generation efficiently eradicates planktonic *S. mutans* by inducing irreversible membrane damage. Simultaneously, the focused thermal stress and physical interfacial interactions effectively dismantle the protective EPS matrix, leading to thorough disintegration and dispersion of mature biofilms. Furthermore, FWA NPs demonstrate remarkable physiological compatibility and negligible toxicity toward mammalian cells, confirming that their robust antimicrobial efficacy is precisely driven by the on-demand photothermal effect rather than non-specific chemical toxicity. By synergistically integrating structural advantages with highly efficient energy conversion and excellent biosafety, this rationally designed nanoplatform provides a highly effective, non-antibiotic therapeutic paradigm for the localized management of biofilm-associated oral infections, and offers a promising strategy to mitigate the growing threat of antimicrobial resistance.

## Data Availability

The original contributions presented in the study are included in the article/**Supplementary Material**. Further inquiries can be directed to the corresponding authors.
