# Supplementary material for: A biomimetic urchin-like photothermal nanoplatform for the efficient eradication of Streptococcus mutans and biofilm dispersion
Source: Front Cell Infect Microbiol. 2026 Sep 3;16:1917604. doi: 10.3389/fcimb.2026.1917604 (PMC13581847; doi:10.3389/fcimb.2026.1917604)
Supplement: Supplementary file 1 [file DataSheet1.pdf]

A Biomimetic Urchin-like Photothermal Nanoplatform for the Efficient Eradication of *Streptococcus mutans* and Biofilm Dispersion

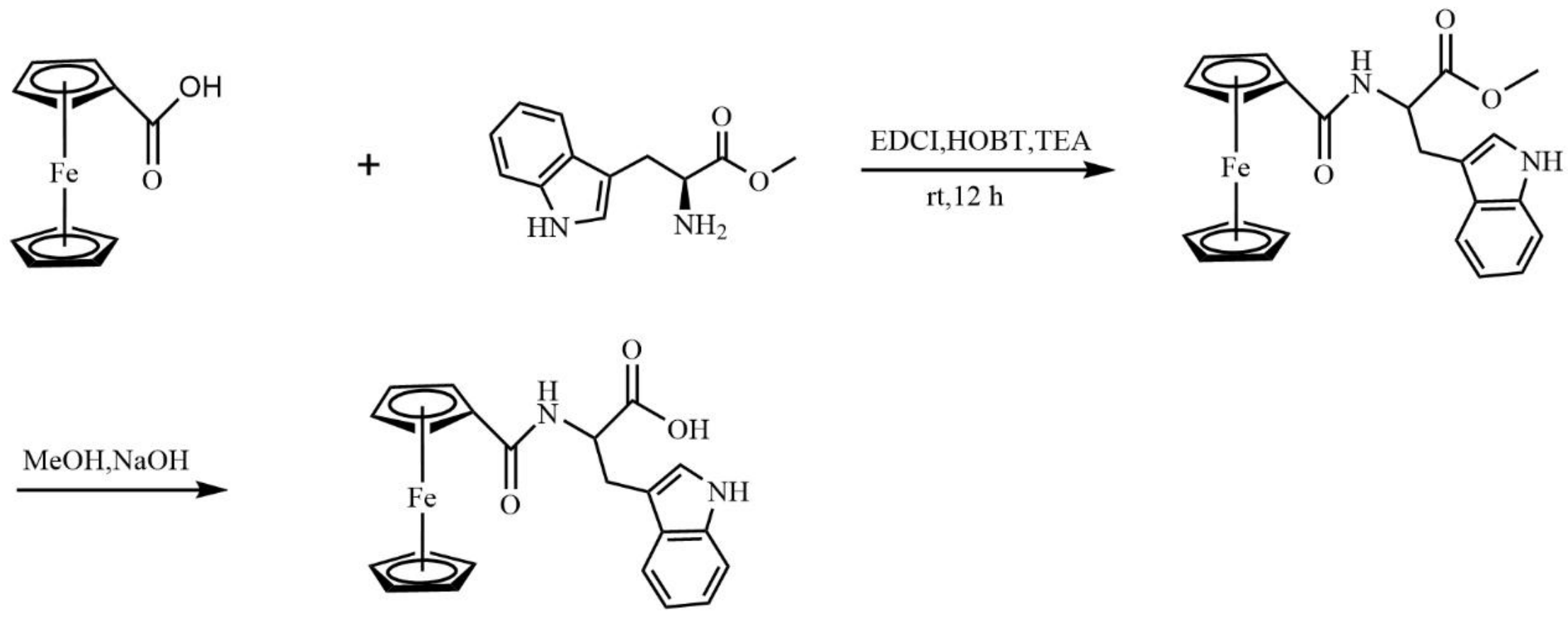

Figure S1. Synthesis route of Fc-W.

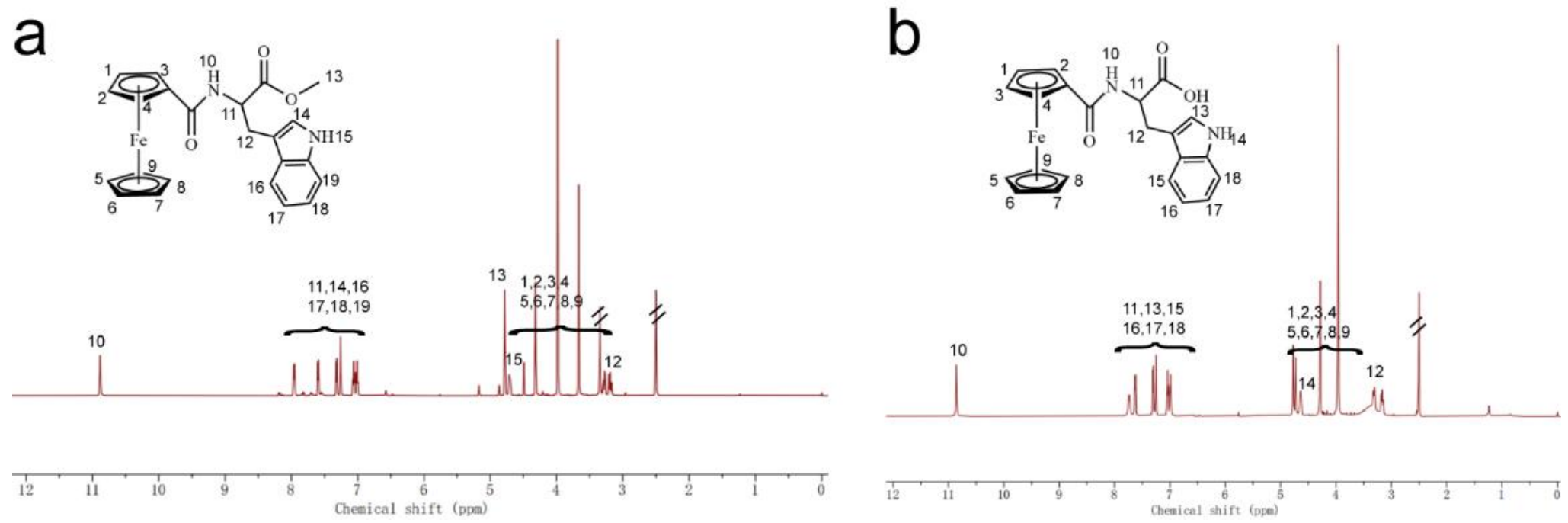

Figure S2. 1H NMR spectrum of Fc-W-Ome (a) and Fc-W (b) in DMSO-*d6*, 400 MHz, 25 °C.
